# Pan-European Sarcoma Trials: Moving Forward in a Climate of Increasing Economic and Regulatory Pressure

**DOI:** 10.1155/2007/76405

**Published:** 2007-06-17

**Authors:** Dorothe Carrle, Tobias Dantonello, Stefan S. Bielack

**Affiliations:** ^1^Klinikum Stuttgart, Olgahospital, Paediatrics 5 (Oncology, Haematology, Immunology), D-70176 Stuttgart, Germany; ^2^Department of Paediatric Hematology and Oncology, University Children's Hospital Muenster, D-48149 Muenster, Germany

## Abstract

Advances in sarcoma treatment are largely based on investigator-initiated, multicentric and interdisciplinary clinical trials. The EU's Good Clinical Practice Directive 2001/20/EC, effective since 2004, was meant to harmonize the conditions for clinical trials across Europe, but, instead, the challenge of initiating and running multinational, noncommercial clinical trials has become greater than ever. Institutions participating in existing noncommercial Pan-European studies are struggling to cope with increased administrative and financial burdens, and few new studies are initiated any more. The aim of a conference entitled “Pan-European Sarcoma Trials: Moving Forward in a Climate of Increasing Economic and Regulatory Pressure,” held in Stuttgart, Germany, 30 November–2 December 2006 as part of the European Science Foundation's ECT-program, was not only to provide an overview of currently active and planned multinational studies on osteo-, Ewing's, and soft tissue sarcoma, but also to draw on areas of synergy between various established sarcoma groups in Europe to define plausible survival strategies for collaborative, interdisciplinary, patient-oriented research.

## 1. BACKGROUND

Since 1 May 2004, the date from which institutions conducting clinical trials in EU member states were obliged to have the laws and administrative procedures in place to comply with Good Clinical Practice under EU Directive 2001/20/EC, the challenge of instigating and running noncommercial clinical trials has been greater than ever. Institutions performing existing noncommercial European trials are struggling to cope with the increased administrative and financial burden caused by the new legislation, and there is a real danger that the onerous set-up criteria combined with increased costs will cause fewer new studies to be initiated. These issues were taken up in a conference initiated by the three sarcoma study groups of the German Society of Paediatric Oncology and Hematology, GPOH, organised by COSS, the Cooperative Osteosarcoma Study Group with support from the European Science Foundation ECT-Programme. Its title “Pan-European Sarcoma Trials: Moving Forward in a Climate of Increasing Economic and Regulatory Pressure” (Stuttgart, Germany, 30 November–2 December 2006) served as motto for more than two hundred investigators from 19 countries who are—or aspire to be—involved in the day-to-day management and/or implementation of Pan-European clinical trials at an institutional level who gathered in Stuttgart for a mixture of plenum presentations and interactive discussion sessions.

The aim of the conference was not only to provide an overview of currently active and planned multinational sarcoma studies (osteo-, Ewing's, and soft tissue) in Europe, but also to draw on areas of synergy between various established sarcoma groups in Europe to define a plausible survival strategy and ensure that joined-up research continues in the future.

## 2. FIRST SESSION—BRINGING QUALITY INTO LIFE: VIEWS AND PERSPECTIVES IN SARCOMA PATIENTS

The importance of taking into account not just survival data in clinical trials but also the patient and his/her family's perspective on quality of life was demonstrated during the quality of life session discussing different approaches to evaluate QOL data.

This session culminated in a tour of the poster exhibition “Bringing Medicine to Life.” Proof of the relevance of this topic and the impact of the presentation is best provided by the fact that a full-page article on the art project was published in *Lancet Oncology* [1].

## 3. SECOND SESSION—THE REGULATORY AND ECONOMIC ENVIRONMENT FOR CLINICAL TRIALS IN EUROPE

In the introduction to this session, S. Bielack, Stuttgart, Germany (ECT-Project Leader, EURAMOS) outlined the need for a collaborative approach in establishing and running international sarcoma trials. Using graphic examples, he demonstrated how the reality of meeting the requirements to implement the EU-Directive 2001/20/EC at a German national level contradicts the good intentions of the politicians and law makers to facilitate and support clinical research. He emphasised that the practical consequences of implementing the new legislation is a source of major concern, especially with regard to legal, financial, and workload aspects.

In her talk “Challenges presented by applying current regulations to the day to day running of trials,” K. Pritchard-Jones (Chairperson, SIOP Europe Clinical Trials Committee), London, UK, shared her insight into the impact of the EU-Directive not distinguishing between commercial and academic trials. During the discussion, it became clear that due to the diversity of national interpretation of the legislation and subsequently different implementations of it into national law, the Directive has not only plainly failed to harmonise the conduct of clinical trials throughout Europe but has also raised major obstacles for the continuance of international collaborations which have functioned well until this point in time.

In presenting the regulators' view of how the EU-Directive was implemented into national law in Germany, C. Steffen, Bonn, Germany (Head, Clinical Trials and GCP Inspections Unit, Federal Institute for Drugs and Medical Devices) gave examples of how as a consequence of having to apply strict “one size fits all” procedures to all clinical trials, his institution is being flooded with—in many cases needless—SAE reports. This “overload” situation hinders intelligent pharmacovigilance. Following Dr. Steffen's presentation, the urgent need for an adaptation of the regulatory procedures to the different needs encountered with different types of trials was discussed.

In his talk “Interpretation and implementation of EU legislation at the national level—the paediatric clinician's perspective,” H. Jürgens, Muenster, Germany (Past-President, German Society of Paediatric Oncology and Haematology and Chairman of the EURO-Ewing trial) explained how the conduct of clinical trials has contributed to the provision of a guaranteed (best) standard of treatment in the care of individual paediatric cancer patients. In taking the audience through a risk versus benefit evaluation of the EU-legislation and its implementation at a national level, he presented pharmacovigilance and professionalisation of research as positive effects, but the huge bureaucratic and financial burdens as negative factors which constitute a major threat to the continued delivery of best quality-standard care. In the lively discussion which followed his risk/benefit analysis, it emerged that a formal centralisation and standardisation at the professional society level might provide a possible partial solution to some problems.

In his talk “Licensing and availability of standard drugs in paediatric oncology—the TEDDY perspective,” P. Paolucci, Modena, Italy (Chairmen, TEDDY (the Task Force in Europe for Drug Development for Young)) presented the current situation from the angle of an organisation whose aim is to build research capacity in the development of paediatric medicines and promote the safe and effective use of existing medicines in children. He stressed the clinical and ethical importance of being able to evaluate paediatric drugs, particularly in view of the current situation with the off-label use of many drugs, the lack of paediatric formulations, and the risk of withdrawal from the market. He suggested different strategies in order to achieve quality, efficacy, and safety of paediatric drugs within these different situations. He also stated that overcautious regulations for marketing authorization requirements counteract the original intention of protecting the interest of the patients and jeopardize the possibility to provide optimum care. The need to build on existing frameworks and work within a multilevel network was made clear during the discussion.

In her talk “Coordination of funding at the Pan-European level—the EUROCORES ECT Programme,” M. Resnicoff, Strassbourg, France (Coordinator, EUROCORES ECT Programme in Medical Sciences) provided a concise outline of the structure and the aims of the EUROCORES ECT Programme. She illustrated how the European Science Foundation (ESF) provides a platform for its member organisations in order to promote research at the European level.

The need for an ongoing dialogue between funders and the researchers who implement Pan-European clinical trials was the key point to come out of the talk “Support from charitable organisations in Germany” presented by F. Kohlhuber, Bonn, Germany (Project Aid Directorate, Deutsche Krebshilfe). He explained that the increased financial and administrative burden as a consequence of the implementation of the EU-Directive accounts for the expanding gap between a limited funding budget and the requirements of research, therefore contributing to a decline in the number of investigator-initiated clinical trials.

In the roundtable discussion, “Balancing the needs of patient-orientated clinical research with the demands of the regulatory environment,” the regulatory obstacles to effective Pan-European collaborations within the various sarcoma groups were discussed. It transpired that—depending on how the EU-Directive has been implemented on a national level—the new legislation has resulted in both facilitating and restricting research, with various degrees of complexity. The discussion then focused on exploring solutions for unresolved issues such as the legal requirement of sponsorship. It was agreed that different national interpretations of the Directive should not hinder Pan-European collaboration on randomised trials, nor obscure the achievement and ongoing need for ensuring a guaranteed standard of care. This was considered especially important in rare diseases, where randomised trials are not feasible. The responsibility of the health insurance companies to prevent a decline in the quality of standard care was also discussed. During this lively and at times heated discussion, it became clear that the session on the regulatory and economic environment had been a mutual learning experience which had provided an excellent opportunity to gain new insights into the perspectives of others.

## 4. THIRD SESSION—OSTEOSARCOMA

The aim of this session was to update the participants of the conference with recent results from multinational trials on the most frequent of the bone sarcomas, to give an update about the current status of the Pan-European/Transatlantic EURAMOS study, to explore ways of expanding the EURAMOS-network to additional European countries, and to develop and foster links with other European bone tumor networks, such as EuroBoNet.

*Skip metastases are not associated with a dismal prognosis, L. Kager, Vienna, Austria*

The prognostic implication of skip metastases in osteosarcoma was retrospectively analysed in patients registered in the neoadjuvant Cooperative Osteosarcoma Study Group studies. It was shown that synchronous regional bone metastases (skip metastases) are rare in osteosarcoma, and preoperative detection relies on appropriate diagnostic imaging. Previously it was believed that patients with skip metastases had an extremely poor prognosis. There, it was shown that aggressive multimodal therapy holds the promise to achieve prolonged survival, especially in patients in whom these metastases occur within the same bone as the primary lesion and whose tumors respond well to chemotherapy [2].

*Dose intensity in osteosarcoma therapy: does it matter? COSS: results from a retrospective analysis of 917 patients, S. Bielack, Stuttgart, Germany*

The possible prognostic relevance of dose intensity in the treatment of osteosarcoma according to several consecutive COSS protocols was retrospectively analysed. In an overall setting of intensive multidrug treatment of osteosarcoma, it could not be proved that a higher dose intensity correlated with better outcomes [3].

*EOI: results from a prospective trial of doxorubicin/cisplatin +/− G-CSF, I. Lewis, Leeds, UK*

The analysis of EOI data did not show a survival benefit for increasing received dose or dose intensity in the context of a two-drug regimen with cisplatin and doxorubicin. The hypothesis that increasing dose intensity may improve survival in osteosarcoma could not be proven. There was no clear evidence of preoperative dose or dose-intensity influencing histologic response [4].

*Updated results of the prospective multicenter trial COSS-96, S. Bielack, Stuttgart, Germany*

Evaluation of a risk-adapted osteosarcoma chemotherapy was the aim of the COSS-96 trial. A four-drug chemotherapy was found highly effective against osteosarcoma. Additional findings were that a long treatment duration may be needed even for presumed low-risk patients. The outcome of high-risk patients remained poor despite salvage treatment. COSS-96 led the COSS group to realize that international collaboration on a much broader level would be required to explore questions such as the potential role of salvage regimens for osteosarcoma and formed the basis for the group's commitment to ECT-EURAMOS [5].

*The European and American osteosarcoma study EURAMOS-1, M. Sydes, London, UK, D. Carrle, Stuttgart, Germany, J. Whelan, London, UK, S. Smeland, Oslo, Norway N. Marina, Stanford, USA, S. Bielack, Stuttgart, Germany, A. Zoubek, Vienna, Austria, A. Holliday, London, UK, J. Stary, Prague, Czech Republic, W. Wozniak, Warsaw, Poland*

The recruitment update from the coordinating data center and progress reports from the collaborating groups COSS, EOI, SSG, and COG were followed by brief reports on special national issues of the participating countries. It emerged that progress was made in that a big Swiss medical oncology center managed to resolve the non-fault insurance issue, otherwise an ongoing issue in Switzerland. Thanks to their huge efforts, the paediatric oncologists in Austria finally overcame the sponsorship issue—an issue still unresolved for the nonpaediatric Austrian oncologists. Requests for participation in EURAMOS from other groups and countries have resulted in the production of an application procedure for applicant countries, which was presented. EURAMOS was presented as an example of a well-functioning Pan-European and American collaboration, while allowing space to maintain the individual profile of each collaborating study group.

*EURAMOS networking amongst osteosarcoma groups*

*EURO-B.O.S.S.: standardised treatment for older patients with osteosarcoma, S. Ferrari, Bologna, Italy*

The outline and preliminary results of a collaborative project involving the three European study groups ISG, SSG, and COSS for patients over 40 years with osteosarcoma and other spindle cell bone sarcoma were presented.

*The European Relapsed Osteosarcoma Registry (EURELOS), C. Int-Veen, Stuttgart, Germany*

EURELOS, a much needed database for relapsed osteosarcoma, another collaboration project between ISG, SSG, and COSS has recently started recruitment.

*Networking clinical osteosarcoma trials with basic research in the EuroBoNet work package, H. Bürger, Muenster, Germany*

EuroBoNet (European network to promote research into uncommon cancers in adults and children: pathology, biology, and genetics of bone tumours network of excellence) is a collaborative programme intended to contribute to obtaining molecular portraits of bone tumours and to allow investigations of specific hypothesis-driven approaches. This would lead to further understanding and identification of markers for malignant transformation and/or progression, as well as identification of therapeutic targets. It is a powerful instrument intended to overcome the fragmentation of the European research landscape with the objective to strengthen European excellence and combine multidisciplinary expertise of pathologist, biologists, and oncologist. Major goals are integration, dissemination of knowledge, and excellence in combined research. The EURAMOS group was called upon to support this effort by networking with EuroBoNet, for example by providing tumour samples for expression array research.

## 5. FOURTH SESSION—INTERGROUP PROJECTS AND STRATEGIES

*Pharmacovigilance in sarcoma trials, T. Butterfass-Bahloul, Muenster, Germany*

Through the process of establishing a functioning pharmacovigilance report system, the EURAMOS intergroup safety desk has gained valuable experience which was shared in order to serve other groups in setting up their own safety reporting systems. The complexities associated with the establishment of a Pan-European safety desk became obvious, arguing for centralisation of such efforts and networking between trials.

*Assessing quality of life in sarcoma trials, G. Calaminus, Duesseldorf, Germany*

The different aspects of quality of life assessment in sarcoma patients were presented. EURAMOS might serve as a model for how to integrate a quality of life assessment project into a trial aiming to optimise treatment strategies in sarcomas.

*Functional impact of surgery on sarcoma patients, C. Gebert, Muenster, Germany*

C. Gebert gave a concise overview on the current surgical approaches for sarcomas and explored if the functional outcome of surgery is determined by the tumour rather than by the surgical method.

*State of the art in surgical therapy of lung metastases, K. Diemel, Grosshansdorf, Germany*

The surgical management of pulmonary metastases with its opportunities and pitfalls were presented in an illustrative way which helped to raise awareness of an adequate approach for the local treatment of pulmonary metastases.

*Comparison of treatment concepts for extraosseous Ewing's sarcoma between soft-tissue and bone sarcoma trials, R. Ladenstein, Vienna, Austria*

An analysis of the therapeutic strategies for extraosseous Ewing's sarcomas within two German Society of Paediatric Oncology and Haematology (GPOH) Cooperative Study Group concepts identified favourable disease factors, limits of the analyses being diverging approaches with regards to tumor assessment and to therapy.

*Late-effects surveillance system (LESS), M. Paulides, Erlangen, Germany*

Results of a prospective study on late effects, performed in the context of a follow-up network for sarcoma patients, were presented. The network was set up in order to standardise and optimise the follow-up and to register major sequelae with simple and sensitive methods.

Results of pilot studies were integrated into the follow-up programs used by EURAMOS-1 and other current sarcoma trials.

## 6. FIFTH SESSION—SOFT-TISSUE SARCOMA

*European challenges in establishing a Pan-European protocol for rhabdomyosarcoma, M. Stevens, Bristol, UK*

*Implementation of European regulations at a national level: barriers to establishing a Pan-European protocol for localised rhabdomyosarcoma, E. Koscielniak, Stuttgart, Germany*

The obvious advantages of conducting trials in European collaborative networks (similar as for EURAMOS-1, faster recruitment of patients, faster therapeutic progress, and improved cooperation and networking between trial groups) was outlined. However the challenges associated with the implementation of the EU-Directive 2001/20/EC jeopardize the conduction of a joint intergroup study.

*Adjuvant chemotherapy in synovial sarcoma and other non-RMS soft-tissue sarcoma: a yet to be resolved controversial question, I. Brecht, Stuttgart, Germany*

Outcomes in young patients with synovial sarcoma treated with intensive multimodal therapy appear to be very promising. It was described that it is only possible to learn more about important treatment questions, for example, the role of adjuvant chemotherapy, in uncommon diseases as non-RMS soft-tissue sarcoma in multinational studies—simply due to the rarity of these tumours in young patients [6].

*Treatment of metastatic soft-tissue sarcoma within the CWS group. Results of the CWS-96 IV study, T. Klingebiel, Frankfurt, Germany*

In this trial, maintenance chemotherapy appeared to lead to better results than high-dose chemotherapy with stem-cell rescue. The presentation showed that not only will the current standard treatment of children with soft-tissue sarcoma suffer if collaborative intergroup studies are impossible, but exciting new developments such as metronomic treatment may not be tested further in randomised trials too.

*Results of the randomised study for localised “high-risk” rhabdomyosarcoma. Report of the CWS-96 and ICG-96 studies, T. Dantonello, Stuttgart, Germany*

The cooperative trials CWS-96 and ICG-96 were presented as examples for a potential solution in the current situation for European soft-tissue sarcoma trials: it may be necessary—and this is certainly not ideal—to return to the level of networking and cooperation achieved in the 1990s: the carrying out of different randomised studies according to a consensus about standard treatment with the use of similar stratifications. It was stressed that this is by no means an ideal solution, but it is at least better than bringing studies to a halt.

*Innovative radiation methods and their role in the treatment of children with soft-tissue sarcoma, A. Schuck, Muenster, Germany*

Different innovative radiation techniques (e.g., proton beam, intensity-modulated, stereotactic) with their individual pros and cons were described. It emerged that these new methods may be useful especially for children with soft-tissue sarcoma due to the young age and sensitive involved sites of the affected patients. It will however be necessary to give these techniques opportunities to evaluate them further in larger cohorts.

*How to realise common European biological research projects for soft-tissue sarcoma within the European Soft-Tissue Sarcoma Study Group, A. Rosolen, Padova, Italy*

The challenges in the realisation of common European biological research projects in soft-tissue sarcoma were explained. Basically, they resemble those of the EuroBoNet, but there is currently no finance for setting-up a similar structure.

*Clinical relevance of molecular diagnosis in rhabdomyosarcoma. Retrospective analysis of the CWS studies, S. Stegmaier, Stuttgart, Germany*

The largest analysis to date regarding the prognostic impact of different fusion types in alveolar rhabdomyosarcoma was presented. It was explained that soft-tissue sarcomas offer interesting molecular research options due to the frequent genetic alterations in these tumours and it would therefore be regrettable if these research opportunities would not be utilised. It was shown how previous studies in smaller samples lead to wrong conclusions. Thus, due to the relative rarity and heterogeneity of these diseases, meaningful biologic research projects require Pan-European cooperation.

### 6.1. Discussion and summary

In the session on soft-tissue sarcoma, it became clear that the problem scenario outlined by Kathy Pritchard-Jones in her talk “Challenges presented by applying current regulations to the day-to-day running of clinical trials” is already reality which is threatening the efficacy, motivation, and long-term survival of established and experienced European sarcoma groups. This predicament is primarily due to different implementations of the clinical trials directive at the individual country level; while some countries have interpreted the legislation to mean that noncommercial academic trials and trials prescribing the currently best available treatment do not have to meet the same compliance criteria as industry-sponsored studies investigating new drugs, others have interpreted it to mean that one should not distinguish between academic and commercial trials at all.

The participants of the meeting were in full agreement that if paediatric cancer patients were to be treated outside clinical trials, the standard of care and the cure rate would suffer. This is particularly valid in a group of very heterogeneous and complex diseases like paediatric soft-tissue sarcoma, which frequently affect very young children and expose them to intense multimodal treatment.

The relevance of the European soft-tissue sarcoma group's problems in overcoming the regulatory hurdles set up by the current European legislation is emphasized by the fact that the experience with the planned Pan-European soft-tissue sarcoma trial was highlighted in recent articles from *Nature Medicine*, which focused on the situation of clinical trials in Europe [7, 8].

## 7. SIXTH SESSION—EWING'S SARCOMA

*Basic requirements in the conventional pathologic workup of Ewing tumours, response evaluation, G. Köhler, Muenster, Germany, and P. Hogendoorn, Leiden, The Netherlands*

In a clear and concise presentation, the requirements of the pathologic workup essential for diagnosis and response evaluation were summarized.

*Current initiatives, targets and markers in Ewing's sarcoma biology, H. Kovar, Vienna, Austria*

It was explained how urgently reliable prognostic markers and novel targeted treatment approaches are required in Ewing tumors. Possible ways of how these markers and targets could be identified were presented with regard to lab investigation and collaborative research in European community funded initiatives focussing on Ewing's sarcoma. It was challenged that overlaps should be avoided in the different initiatives and how potential synergies could be used. Intermediate results from the accompanying biological studies in the ongoing EURO-Ewing trial and the lessons to be learned from these projects were demonstrated.

*EICESS 92—Global results and results according to local therapy, J. Whelan, London, UK*

The results of the collaborative EICESS trial as example of a large international trial in a rare disease were presented, showing that there was no differences in the randomised treatment arm with regard to overall survival. The different treatment approaches within the participating groups of the intergroup trial resulting in moderate survival differences were highlighted. The trial showed that a greater use of surgery was associated with survival advantages and stressed the standardisation of local treatment.

*EICESS 92—Results according to age and institution, M. Paulussen, Basel, Switzerland*

The previously divergent results of potential advantages of treatment of adolescents/young adults according to paediatric protocols were illustrated. The EICESS 92 trial as uniform protocol for children, adolescents, and adults provided a unique opportunity to study outcomes according to age and institution. It showed less differences between paediatric and nonpaediatric institutions as compared with previous studies and no differences any longer in patients with localised disease.

*Interim report on EURO-E.W.I.N.G.99, H. Jürgens, Muenster, Germany*

EURO-E.W.I.N.G.99 is a Pan-European intergroup trial initiated long before any attempt to harmonise GCP regulations across Europe. As of October 2006, nearly 2000 patients could be recruited into the trial and randomisation compliance was still improving. Recruitment thus currently exceeds the expected numbers in all treatment arms. Final results are yet to be awaited due to the ongoing nature of the trial, but response to neoadjuvant chemotherapy seems to be superior to the previous CESS and EICESS studies.

*EURO-E.W.I.N.G.99—R3 results, R. Ladenstein, Vienna, Austria*

Results of the treatment arm for Ewing tumours with metastases to bone, bone marrow, and multifocal sites were presented, stressing the role of the various high-dose treatments and the continuously poor prognosis for these patients.

*Ewing tumours in infants, H. van den Berg, Amsterdam, The Netherlands*

The treatment results of 14 infants with Ewing's sarcoma treated in one of the consecutive CESS, EICESS, and EURO-E.W.I.N.G.99s trials were presented, demonstrating that—in contrast to the literature—the majority of tumours were pPNET, the sarcomas were entirely axial, and overall survival was comparable to older children.

*The value of FDG-PET in staging and response evaluation, U. Dirksen, Muenster, Germany*

The value of PET in Ewing sarcoma staging and its possible role for evaluation of response as new prognostic marker were explained with emphasis on the need to conduct further studies in this topic. Already now, the role of PET scans is established in Ewing's sarcoma due to its superior sensibility in detecting bone lesions compared to classic bone scans [9].

*The value of treosulfan in the treatment of high-risk Ewing tumours, U. Dirksen, Muenster, Germany*

The role of treosulfan in cancer treatment was reviewed, and promising results of a study exploring its use in paediatric patients were demonstrated. Treosulfan shows high cytotoxic activity against Ewing cells and may be a promising agent, but its efficacy regarding the treatment of Ewing's sarcoma currently remains to be proven. The safety profile of treosulfan is however acceptable.

### 7.1. Discussion and summary

The EURO-E.W.I.N.G. study started in 1999 and is still ongoing. It was explained that the trial has recently been prolonged to achieve the expected patient numbers in certain subgroups and that the study was only possible in the first place because it was started prior to the implementation of EU-Directive 20/2001/EC. It became clear during the discussion that rather than facing the arduous struggle with the bureaucracy associated with the Directive, researchers are seeking to prolong existing studies. While this is understandable, these prolongations could well be regarded as a deceleration of clinical research.

## 8. CONCLUSIONS AND RECOMMENDATIONS FOR FURTHER ACTION

Initiated by the ECT-EURAMOS group and lead by COSS, sarcoma study groups from across Europe gathered in Stuttgart to share scientific knowledge, to intensify their Pan-European and Transatlantic collaboration, and to approach the administrative, regulatory, and financial challenges brought along with the European Clinical Trials Directive.

The concept of stepping out of the scientific community and approaching representatives from governments, regulatory authorities, and funding organisations in order to emphasize common challenges and to discuss constructive solutions proved successful. Coverage regarding both the session on the regulatory and economic environment for clinical trials in Europe meeting itself [10] and the specific problems of establishing a Pan-European paediatric soft-tissue sarcoma study under legislation driven by EU-Directive 2001/20/EC (discussed at the meeting, see above, second and fifth sessions, highlighted in *Nature Medicine* [7, 8]) can be seen as direct proof of the raised awareness through this meeting.

The intensive discussion resulted in an input on the “Draft guidance on “specific modalities” for noncommercial clinical trials referred to in Commission Directive 2005/28/EC laying down the principles and detailed guidelines for good clinical practice” by SIOP Europe [11]. The conference also facilitated the exchange of ideas and experiences and allowed for synergic effects. For example, the effort put in the development of a meanwhile well-established, well-functioning, internationally recognized GCP-conformal pharmacovigilance system resulted in recognizing it as a model for other groups. The awareness that treatment outside clinical trials will lead to a decline in cure rates, particularly in a group of very heterogeneous and complex diseases such as sarcomas, was well perceived. In rare sarcoma subtypes, where prospective, randomised clinical trials are unfeasible due to lack of numbers, alternative intergroup strategies need to be pursued.
